# Risk Factors for Gestational Diabetes Mellitus in a Large Population of Women Living in Spain: Implications for Preventative Strategies

**DOI:** 10.1155/2012/312529

**Published:** 2012-04-12

**Authors:** Ana M. Ramos-Leví, Natalia Pérez-Ferre, M. Dolores Fernández, Laura del Valle, Elena Bordiu, Ana Rosa Bedia, Miguel A. Herraiz, M. José Torrejón, Alfonso L. Calle-Pascual

**Affiliations:** ^1^Department of Endocrinology and Nutrition, Hospital Clínico San Carlos, Professor Martín Lagos Street s/n, 28040 Madrid, Spain; ^2^Department of Laboratory Analysis, Hospital Clínico San Carlos, Professor Martín Lagos Street s/n, 28040 Madrid, Spain; ^3^Department of Gynecology and Obstetrics, Hospital Clínico San Carlos, Professor Martín Lagos Street s/n, 28040 Madrid, Spain

## Abstract

The aim of this study is to establish a risk appraisal model for GDM by identifying modifiable factors that can help predict the risk of GDM in a large population of 2194 women living in Spain. They were recruited between 2009-2010 when screening for GDM was performed. Participants completed a questionnaire on socio-demographic, anthropomorphic and behavioral characteristics, and reproductive and medical history. A total of 213 (9.7%) women were diagnosed as having GDM. Age, pregestational body weight (BW) and body mass index (BMI), and number of events of medical, obstetric and family history were significantly associated with GDM. After logistic regression model, biscuits and pastries intake <4 times/week, red and processed meats intake <6 servings/week, sugared drinks <4 servings/week, light walking >30 minutes/day, and 30 minutes/day of sports at least 2 days/week, compared with opposite consumption, was associated with less GDM risk. Our study identified several pregestational modifiable lifestyle risk factors associated with an increase in the risk of developing GDM. This may represent a promising approach for the prevention of GDM and subsequent complications. Further intervention studies are needed to evaluate if this appraisal model of risk calculation can be useful for prevention and treatment of GDM.

## 1. Introduction

Gestational diabetes mellitus (GDM) has been defined for many years as any degree of glucose intolerance with onset or first recognition during pregnancy [1]. According to the hyperglycemia and adverse pregnancy outcomes (HAPO study) [2], a large-scale multinational epidemiologic study, the risk of adverse maternal, fetal, and neonatal outcomes continuously increases as a function of maternal glycemia at 24–28 weeks of gestation. There was no threshold for most of these complications, but preventing and early identifying GDM is a growing health concern.

Because the prevalence of GDM is increasing in a similar way to the ongoing epidemic of obesity and type 2 diabetes in women of child-bearing age [3], understanding the significance of risk factors becomes of highlighted importance. Nonmodifiable risk factors such as past history of GDM and increasing maternal age have been identified [4, 5]. However, the impact of diet and lifestyle modifiable risk factors has not yet been systematically synthesized [6, 7]; they can be different in countries of the Mediterranean area, and only preliminary data are known [8].

The aim of this study is to establish a risk appraisal model for GDM by identifying modifiable factors that can help predict the risk of GDM in a large population of women living in Spain and target early intervention, at the time of screening for GDM, with the potential of preventing the development of or ameliorating GDM.

## 2. Population, Research Design, and Methods

Participants were recruited from women attending prenatal care at our Hospital during the years 2009-2010, at 24–28 weeks of gestation, when screening for GDM was performed. A total of 2194 women were invited to participate and gave their written informed consent. The study was approved by the Ethical Committee of the Hospital Clínico San Carlos and was carried out following the principles expressed in the Helsinki Declaration.  Table 1 shows a summary of the demographic and anthropomorphic characteristics of the study population, according to the positive or negative diagnosis of GDM.

Women were screened for GDM based on the two-step American Diabetes Association recommendations: at week 24–28 of gestation, women with no previous history of diabetes mellitus were assessed, after a 12-hour fasting and no diet restriction on previous days, via the O'Sullivan test (OS). When plasma glucose levels one hour after glucose load were ≥7.2 mmol/L (≥140 mg/dL), a further 100-gram OGTT was performed, and new glucose levels were measured while fasting and 1, 2, and 3 hours after the intake. GDM was diagnosed according to Coustan and Carpenter criteria.

At enrollment, participants completed a questionnaire with supervision of a trained nurse. These questionnaires were used to gather information on sociodemographic, anthropomorphic and behavioral characteristics, and reproductive and medical histories. The following were stratified in a semiquantitative way: history of smoking habit (never, smoke—to 6 months before pregnancy,—to pregnancy,—currently), pregestational and gestational physical activity (walking frequency, climbing up stairs per day and at least 30-minute moderate intensity sports per week), and pregestational and gestational weekly intake of vegetables, salads, fruit, dried fruits, nuts, blue fish, whole wheat bread, legumes, skimmed dairy products, red and processed meats, sauces, sugared drinks and sodas, juices, biscuits, pastries, alcohol, coffee and water. This questionnaire has been previously reported [9, 10].

Regarding medical history, three groups were analyzed: obstetric history, which included previous abortion, GDM and hypertension; personal medical history, which considered overweight, dyslipidemia, hypertension and altered glucose metabolism; family history, which grouped diabetes, obesity, dyslipidemia and hypertension. Answers were reorganized according to the number of events in each category, and the differences between the GDM and non-GDM groups were analyzed (Table 2).

IBM SPSS program version 19.0 was used for data processing. The relationship between the nonmodifiable factors age, pregestational weight, ethnicity, and personal and family past medical history, and the independent variable GDM, was assessed. The population's lifestyle habits were evaluated, and the chi-square test was used to investigate the existence of possible differences in their association to the characteristic of GDM/non-GDM.

To analyze the effects of the different items of lifestyle habits, a generalized lineal model of binary logistic type was performed. The dependent variable was the diagnosis or not of GDM; reference category was taken as value 0, meaning non-GDM, whilst diagnosis of GDM was taken as value 1. Seventeen items were selected as predictors of GDM: ten related to pregestational intake of nutrients (biscuits and pastries, red and processed meats, fruit, dried fruit and nuts, skimmed dairy products, legumes, whole wheat bread, blue fish, sauces, vegetables and salad); four regarding pregestational consumption of beverages (water, alcohol, sugared drinks and juices and coffee); three related to pregestational physical activity (sports, light walking and climbing up stairs). For each of the previous indicators, intensity was graded according to an ordinary increasing scale with three category levels; low, medium, and high. Missing values were excluded from the analysis. A model of the principal effects of each of the factors considered was chosen, including the intercept. Hybrid method was selected to estimate the different parameters. Type III analysis was used because of the fact that the items considered do not follow any specific arrangement.

To be able to refine and improve the interpretation of the results of the generalized lineal model of binary logistic type, an automatic lineal regression analysis of the model was elaborated. The characteristic of GDM was again considered the outcome, and all of the items previously referred to regarding lifestyle habits were taken as entry predictors. The option of automatic preparation of data was selected; this option may group some of the categories of the items so that the procedure's predictive capacity can be optimized.

## 3. Results

Mean age of the pregnant women enrolled in the study was 31.35 years old [range 13–47]. Diagnosis of GDM was confirmed in 213 (9.7%) women (Table 1). Differences in GDM rate were observed between ethnicities (*P* ≤ 0.05), although it must be pointed out that the number of African and Asian women in the sample was small.

Age, pregestational BW and BMI did not follow a normal distribution (Kolmogorov-Smirnof test, *P* < 0.001 in all cases). Independent samples of Mann-Whitney U Test used to compare the effect of these variables on the diagnosis of GDM showed significant differences between the GDM and non-GDM groups. However, some of these differences were not observed when comparisons according to the different ethnicities were made (Table 1).

The distribution of the number of events related to medical history, according to the different ethnicities, for the GDM and non-GDM groups is shown in Table 2. The chi-square test showed significant differences for some of the comparisons and these were more evident when cases for which events were unknown were excluded. Again, it is worth mentioning the fact that, in some cases, the number of observations was small.

**Figure 1:**
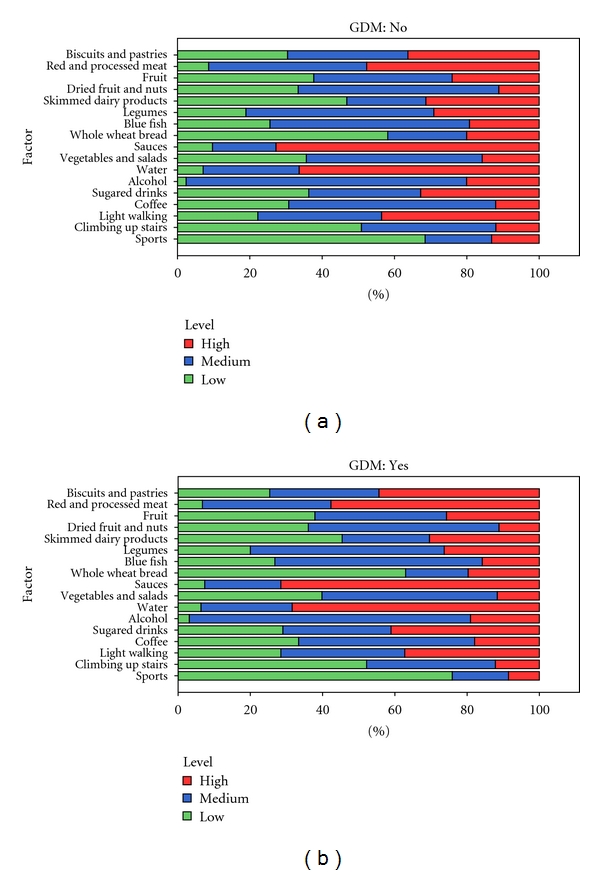
Lifestyle patterns of our study population, according to the diagnosis of GDM. For each of the elements considered, for simplifying purposes, a categorical schematic scale of three levels—low, medium, and high—was elaborated to classify the quantity of intake or practice. The limits varied depending on the factor. Biscuits and pastries <2/week, 2–4/week, >4/week; red and processed meats <3/week, 3–6/week, >6/week; fruit <6/week, 6–12/week, >12/week; dried fruits and nuts 0/week; 1–3/week; >3/week; skimmed dairy products <3/week, 3–6/week, >6/week; legumes <1/week, 1-2/week, >2/week; blue fish <3/week, 3–6/week, >6/week; whole wheat bread <1/week, 1–3/week, >3/week; sauces <2/week, 2–4/week, >4/week; vegetables and salads <6/week, 6–12/week, >12/week; water no, shared, exclusive; alcohol 1–4/day, 4–6/day, >6/day; sugared drinks <2/week, 2–4/week, >4/week; coffee 0-1/day 2-3/day, >3/day; light walking <30 minutes/day, 30–60 minutes/day, >60 minutes/day; climbing up stairs <4/day, 4–16/day, >16/day; sports <2 days/week, 2-3 days/week, >3 days/week. (“<” means less than; “>” means more than).

Figure 1 displays the results of lifestyle patterns according to the diagnosis of GDM. The colored bar represents the distribution of the intake levels or practice frequency of each lifestyle item. Significant differences in the intake of red and processed meats (*P* = 0.023), sugared drinks (*P* = 0.035), and coffee (*P* = 0.022) were found. We observed a trend for differences in biscuits and pastries (*P* = 0.068) and lifestyle habits related to sports and light walking (*P* = 0.073 and *P* = 0.088, resp.). Differences among the remaining factors were not found.

When comparing distributions by pairs, the following significant differences were found: high intake of biscuits and pastries (>4/week), red and processed meats (>6/week), sugared drinks (>4/week), and coffee (>3/day), and low level of practice of sports (<2 days/week) and light walking (<30 minutes/day) was greater amongst GDM. Low intake (<2/week) of sugared drinks was greater in non-GDM.

Logistic regression model is shown in Table 3. The significant coefficients observed were red and processed meats = 3–6 times/week (*B* = −0.423; *P* = 0.015); coffee = 2-3 times/day (*B* = −0.472; *P* = 0.051); sauces = 2–4/week (*B* = 0.394; *P* = 0.058); practice of sports = less than 2 times/week (*B* = 0,676; *P* = 0.038).

**Figure 2:**
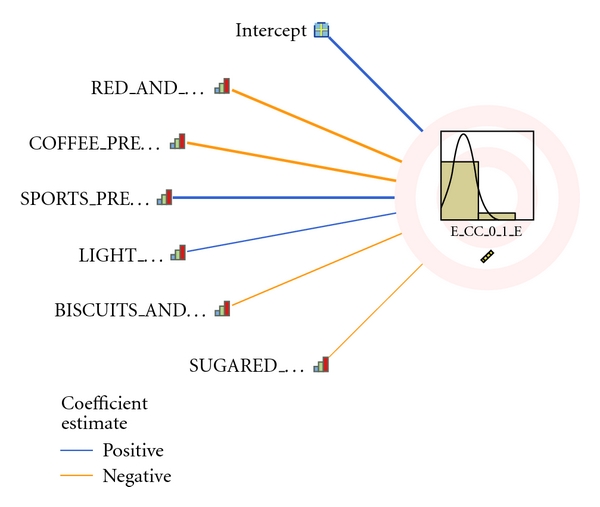
Influence in GDM of each factor according to the automatic lineal regression model. “Positive” means lower risk for GDM and “Negative” means greater risk for GDM. The width of the line is directly proportional to the magnitude of the effect and the significance.

**Figure 3:**
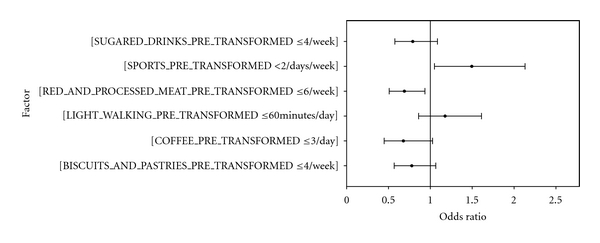
Odds ratio (95% CI) of specific cutoff points of lifestyle factors when applying logistic regression.

Automatic lineal modeling discarded all except the following factors: biscuits and pastries, red and processed meats, sugared drinks, coffee, light walking, and sports. The model reclassified the values as shown in Table 4 in order to optimize the signification level. The direction of the influence of the each factor is sketched in Figure 2. Bearing this fact in mind, the logistic regression using the transformed variables as independent variables was applied. The new equation for GDM = 1 obtained is shown in Table 5. The signification of factors are now biscuits and pastries <4/week (*P* = 0.118), red and processed meats <6/week (*P* = 0.017), sugared drinks <4/week (*P* = 0.146), coffee <3/day (*P* = 0.066), light walking <60 minutes/day (*P* = 0.310), and sports <2 days/week (*P* = 0.026). The corresponding odds ratio (OR) at 95% CI are represented in Figure 3.

## 4. Discussion

The prevalence of GDM in different ethnic populations has been reported between 1 and 17.8% [11, 12]. The number is steadily increasing [13], and it is becoming a growing health concern during the last decade. According to data obtained in this study, the prevalence of GDM was between 2–17%, similar to figures previously reported. Considering this number, the purpose of our study gains importance, as potential nutritional intervention could be carried out to avoid progression of prevalence.

Previous risk factors established for GDM, including prepregnancy BW and BMI, age and history of GDM [14, 15], were confirmed in this study as independent predictors of glucose intolerance.

Regarding the distribution of eating habits, the results show that intake of certain foods can modify the GDM risk. Several published case-control and prospective cohort studies have examined associations of diet with GDM [16–21]. They mostly agreed that diet with low fiber, low complex carbohydrates and high glycemic load was associated with an increased risk of GDM. According to data obtained in this study, differences between the GDM and the non-GDM group in the distribution of the intake of biscuits and pastries, red and processed meats, coffee and sugared drinks were found. The association of these factors to the risk of developing type 2 diabetes is widely known, and because of the increasing prevalence of the analogous GDM, several published studies have begun to study this association. Our data support these previous findings and go one step further in providing new information; they show which types of food are related to higher risk of developing GDM and with what odds. We can then calculate a coefficient of risk based on the level (low, medium, or high) of the factor considered and evaluate how the odds of being diagnosed as GDM are modified.

By using generalized logistic regression and an automatic lineal regression model, we could identify the factors related to GDM. We could further analyze them according to age, weight or ethnicity. This stratification would not be possible for all of the items in the questionnaire because for some of the options data would be insufficient. To be able to assure the influence of these factors despite other circumstances such as age or ethnicity, our study population should be greater, allowing a greater number of women diagnosed with GDM.

If we look at the results regarding coffee intake, although its association with type 2 diabetes has been widely studied [22, 23], there are few papers that consider its role in pregnant women. The systematic review of Van Dam and Hu [23] concluded that habitual coffee consumption was associated with a substantially lower risk of type 2 diabetes, but Wedick et al. [22], on the other hand, could not demonstrate changes in glycemia or insulin sensitivity with coffee consumption. The study by Adeney et al. [24] concluded that moderate prepregnancy caffeinated coffee consumption may have a protective association with GDM. Our study agrees with these previous findings and emphasizes the fact that a higher level of consumption can nullify this protective effect. A possible explanation for this is that, in Spanish population, an increase in coffee intake is associated with an increase in the intake of whole milk and sweets. Prospective studies are still necessary to establish a possible threshold.

Another emerging factor whose influence in GDM is being evaluated is physical activity. Previous data have described that this is one of the strongest predictors contributing to the inverse association to GDM [15, 25–27], and the results of a recent systematic review and meta-analysis demonstrated that greater physical activity before or during early pregnancy is significantly associated with lower risk of GDM, with the magnitude of the association being stronger for prepregnancy physical activity [6]. Our results agree with these previous studies, showing that the less exercise women practiced prior to pregnancy, the more likely they were to develop GDM, and vice versa, the more exercise, the less likely the diagnosis of GDM.

Our study has some limitations that are worth mentioning. Firstly, data regarding information on sociodemographic anthropometric and behavioral characteristics was gathered via self-report in questionnaires. Although these were completed with supervision of a trained nurse, we cannot rule out possible imprecision of pregestational BW and misclassification of food frequency intake and lifestyle habits. This is a common bias in any nutritional epidemiological study, but we assume, however, that bias would occur in a constant and random way, especially because women were unaware of their GDM diagnosis at the time of assessment, and the resulting error would be minimized.

Another possible source of bias is the differential reporting by participant subgroups. For example, women with higher BMI may tend to underreport their food intake, either globally or with specific foods, such as “junk foods.” This introduces a source of confounding that is difficult to account for statistically, although, again, we assume that the magnitude of the error is low, because women were unaware of the OGTT result.

Instructions for women attending prenatal care for GDM screening were the same for all participants, all had no previous history of diabetes mellitus and all glucose determinations were made in the same laboratory. This avoids possible measurement errors and misclassification of GDM.

The fact that information on lifestyle habits was reported to be “pregestational” allows a plausible temporal relationship between behavioral characteristics and GDM, which usually develops after the second trimester.

In conclusion, promoting healthy lifestyle habits among women of reproductive age, such as moderate intake of coffee, low intake of biscuits and pastries, sugared drinks and red and processed meats, and regular physical activity, may represent a promising approach for the prevention of GDM and subsequent complications of children born from pregnancies affected by GDM. Moreover, if we know what a woman's pregestational lifestyle habits are before the screening for GDM, we can predict how the risk is modified, in relation to the population's prevalence. However, further intervention studies are needed to evaluate if this appraisal model of risk calculation can be useful for prevention and treatment of altered glucose metabolism during pregnancy. It is still unknown whether intervention in dietary habits and beginning an exercise routine before pregnancy results in GDM prevention. Further research and cost-effectiveness studies are warranted to establish the possible risk reduction and economic benefit.

## Figures and Tables

**Table 1:** Demographic and anthropomorphic characteristics of the 2194 women enrolled in the study, according to diagnosis of GDM.

Ethnicity		GDM						
		NO			Yes			
		*N*	Mean	SD	*N*	Mean	SD	*P* value
Caucasian Spanish	Age (years)	1068	32.7	5.0	134	35.0	4.3	0.000
	Pregestational body weight (kg)		60.9	10.5		68.5	14.7	0.000
	Pregestational BMI (kg/m^2^)		22.7	3.7		25.7	5.2	0.000
	Gestational body weight (kg)		65.8	10.6		71.4	14.8	0.000
	Gestational BMI (kg/m^2^)		24.6	3.7		26.9	5.0	0.000

Caucasian non-spanish	Age (years)	121	29.0	5.1	13	30.5	4.6	0.264
	Pregestational body weight (kg)		60.8	10.3		73.5	23.2	0.038
	Pregestational BMI (kg/m^2^)		22.4	3.5		27.9	8.1	0.016
	Gestational body weight (kg)		67.2	12.6		72.7	13.3	0.114
	Gestational BMI (kg/m^2^)		24.5	4.5		27.9	5.0	0.041

Hispanic	Age (years)	692	29.1	5.9	54	34.0	4.8	0.000
	Pregestational body weight (kg)		61.3	12.3		68.8	12.4	0.000
	Pregestational BMI (kg/m^2^)		24.3	4.8		27.6	4.9	0.000
	Gestational body weight (kg)		66.5	12.0		70.0	11.1	0.014
	Gestational BMI (kg/m^2^)		26.3	4.6		28.4	4.1	0.001

African	Age (years)	42	29.4	5.7	8	34.0	5.5	0.053
	Pregestational body weight (kg)		67.1	11.6		63.6	8.0	0.436
	Pregestational BMI (kg/m^2^)		25.7	3.4		24.1	2.1	0.324
	Gestational body weight (kg)		70.5	12.1		69.5	8.2	0.900
	Gestational BMI (kg/m^2^)		26.8	3.8		26.0	2.2	0.559

Asian	Age (years)	28	28.2	5.9	2	31.5	0.7	0.588
	Pregestational body weight (kg)		52.5	7.7		·	·	—
	Pregestational BMI (kg/m^2^)		19.9	2.5		·	·	—
	Gestational body weight (kg)		59.1	8.1		52.5	3.5	0.193
	Gestational BMI (kg/m^2^)		22.5	2.3		22.9	·	0.931

Other	Age (years)	30	29.4	7.0	2	31.0	8.5	0.700
	Pregestational body weight (kg)		58.2	8.1		·	·	—
	Pregestational BMI (kg/m^2^)		21.6	2.1		·	·	—
	Gestational body weight (kg)		65.8	10.6		·	·	—
	Gestational BMI (kg/m^2^)		25.0	3.7		·	·	—

**Table 2:** Number of women with gestational, personal and family medical history for the 2194 women enrolled in the study, according to the diagnosis of GDM.

Ethnicity	History	Events	GDM				*P* value
			No		Yes		
			*N*	%	*N*	%	
Caucasian Spanish	Gestational history	None	815	76.4	88	66.2	0.002
		One	233	21.8	37	27.8	
		More than one	13	1.2	7	5.3	
		Unknown	6	0.6	1	0.8	
	Personal Medical history	None	955	89.5	110	82.7	0.028
		One	101	9.5	19	14.3	
		More than one	5	0.5	3	2.3	
		Unknown	6	0.6	1	0.8	
	Family Medical history	None	310	37.5	30	36.1	0.101
		One	276	33.4	19	22.9	
		More than one	236	28.5	33	39.8	
		Unknown	5	0.6	1	1.2	

Caucasian non-Spanish	Gestational history	None	76	62.8	8	61.5	0.158
		One	41	33.9	3	23.1	
		More than one	1	0.8	1	7.7	
		Unknown	3	2.5	1	7.7	
	Personal medical history	None	110	90.9	12	92.3	0.871
		One	6	5.0	1	7.7	
		More than one	1	0.8	0	0	
		Unknown	4	3.3	0	0	
	Family medical history	None	53	54.6	8	72.7	0.399
		One	29	29.9	1	9.1	
		More than one	11	11.3	2	18.2	
		Unknown	4	4.1	0	0	

Hispanic	Gestational history	None	394	57.0	24	44.4	0.024
		One	250	36.2	21	38.9	
		More than one	19	2.7	5	9.3	
		Unknown	28	4.1	4	7.4	
	Personal medical history	None	574	83.1	35	66.0	0.008
		One	80	11.6	10	18.9	
		More than one	7	1.0	2	3.8	
		Unknown	30	4.3	6	11.3	
	Family medical history	None	333	56.1	16	43.2	0.296
		One	156	26.3	12	32.4	
		More than one	75	12.6	5	13.5	
		Unknown	30	5.1	4	10.8	

African	Gestational history	None	23	54.8	5	62.5	0.791
		One	17	40.5	3	37.5	
		More than one	2	4.8	0	0	
		Unknown	0	0	0	0	
	Personal medical history	None	39	92.9	5	62.5	0.015
		One	3	7.1	3	37.5	
		More than one	0	0	0	0	
		Unknown	0	0	0	0	
	Family medical history	None	20	57.1	1	14.3	0.052
		One	13	37.1	4	57.1	
		More than one	2	5.7	2	28.6	
		Unknown	0	0	0	0	

Asian	Gestational history	None	20	71.4	2	100	0.377
		One	8	28.6	0	0	
		More than one	0	0	0	0	
		Unknown	0	0	0	0	
	Personal medical history	None	26	92.9	2	100	0.696
		One	2	7.1	0	0	
		More than one	0	0	0	0	
		Unknown	0	0	0	0	
	Family medical history	None	15	65.2	0	0	—
		One	4	17.4	0	0	
		More than one	4	17.4	0	0	
		Unknown	0	0	0	0	

Other	Gestational history	None	18	64.3	0	0	0.082
		One	7	25.0	2	100	
		More than one	0	0	0	0	
		Unknown	3	10.7	0	0	
	Personal medical history	None	20	71.4	2	100	0.677
		One	5	17.9	0	0	
		More than one	0	0	0	0	
		Unknown	3	10.7	0	0	
	Family medical history	None	10	47.6	0	0	—
		One	3	14.3	0	0	
		More than one	6	28.6	0	0	
		Unknown	2	9.5	0	0	

**Table 3:** Logistic regression equation for GDM = 1 using pregestational lifestyle habits.

−0,3862 ∗ [biscuits and pastries = <2/week] + −0,2925 ∗ [biscuits and pastries = 2–4/week] +
−0,3664 ∗ [red and processed meats = <3/week] + −0,4235 ∗ [red and processed meats = 3–6/week] +
−0,2434 ∗ [fruit = <6/week] + −0,2750 ∗ [fruit = 6–12/week] +
−0,0780 ∗ [dried fruit and nuts = <0/week] + −0,2132 ∗ [dried fruit and nuts = 1–3/week] +
−0,07478 ∗ [skimmed dairy products = <3/week] + 0,1928 ∗ [skimmed dairy products = 3–6/week] +
0,1409 ∗ [legumes = <1/week] + 0,1305 ∗ [legumes = 1-2/week] +
0,0580 ∗ [blue fish = <3/week] + 0,3042 ∗ [blue fish = 3–6/week] +
0,1638 ∗ [whole wheat bread = <1/week] + −0,3230 ∗ [whole wheat bread = 1–3/week] +
−0,2706 ∗ [sauces = <2/week] + 0,3943 ∗ [sauces = 2–4/week] +
0,3967 ∗ [vegetables and salads = <6/week] + 0,3068 ∗ [vegetables and salads = 6–12/week] +
−0,1582 ∗ [water = no] + 0,0288 ∗ [water = shared] +
0,2084 ∗ [alcohol = 1–4/day] + 0,0998 ∗ [alcohol = 4–6/day] +
−0,2761 ∗ [sugared drinks = <2/week] + −0,01169 ∗ [sugared drinks = 2–4/week] +
−0,2931 ∗ [coffee = 0-1/day] + −0,4721 ∗ [coffee = 2-3/day] +
0,2078 ∗ [light walking = <30 minutes/day] + 0,0530 ∗ [light walking = 30–60 minutes/day] +
−0,1544 ∗ [climbing up stairs = <4/day] + −0,1006 ∗ [climbing up stairs = 4–16/day] +
0,6758 ∗ [sports = <2 days/week] + 0,3991 ∗ [sports = 2-3 days/week] + −2,357

**Table 4:** Cutoff points identified by the automatic lineal regression model.

Factor (transformed)	Value = 0	Value = 1
Biscuits and pastries	≤4/week	>4/week
Red and processed meats	≤6/week	>6/week
Sugared drinks	≤4/week	>4/week
Coffee	≤3/day	>3/day
Light walking	≤60 minutes/day	>60 minutes/day
Sports	<2 days/week	≥2 days/week

**Table 5:** Equation for GDM = 1 when applying logistic regression using the transformed variables as independent variables.

−0,2511 ∗ [biscuits and pastries = ≤4/week] +
−0,3717 ∗ [red and processed meats = ≤6/week] +
−0,2351 ∗ [sugared drinks = ≤4/week] +
−0,3885 ∗ [coffee = ≤3/day] +
0,1625 ∗ [light walking = ≤60 minutes/day] +
0,4025 ∗ [sports = <2 days/week] +
+ −1,819
